# Antibiotic Resistance in Lactic Acid Bacteria from Dairy Products in Northern Italy

**DOI:** 10.3390/antibiotics14040375

**Published:** 2025-04-03

**Authors:** Irene Floris, Roberta Battistini, Clara Tramuta, Aitor Garcia-Vozmediano, Noemi Musolino, Giulia Scardino, Chiara Masotti, Beatrice Brusa, Riccardo Orusa, Laura Serracca, Elisabetta Razzuoli, Francesca Martucci, Daniela Manila Bianchi

**Affiliations:** 1 Istituto Zooprofilattico Sperimentale del Piemonte, Liguria e Valle d’Aosta, Via Bologna 148, 10154 Torino, Italy; irene.floris@izsplv.it (I.F.); clara.tramuta@izsplv.it (C.T.); aitor.garciavozmediano@izsplv.it (A.G.-V.); nmusolino@asl.at.it (N.M.); giulia.scardino@asl.novara.it (G.S.); chiara.masotti@izsplv.it (C.M.); beatrice.brusa@izsplv.it (B.B.); riccardo.orusa@izsplv.it (R.O.); laura.serracca@izsplv.it (L.S.); elisabetta.razzuoli@izsplv.it (E.R.); francesca.martucci@izsplv.it (F.M.); manila.bianchi@izsplv.it (D.M.B.); 2 Azienda Sanitaria Locale di Asti, Via Conte Verde 125, 14100 Asti, Italy; 3 Azienda Sanitaria Locale Asl Novara, Viale Roma 7, 28100 Novara, Italy

**Keywords:** antimicrobial resistance, lactic acid bacteria, raw milk, antibiotic resistance gene, PCR

## Abstract

**Background**: The spread of antibiotic resistance genes (ARGs) from the food chain is a significant public health concern. Dairy products from raw milk containing lactic acid bacteria (LAB) resistant to antimicrobials may serve as vectors for the transfer of resistance to commensal or potentially pathogenic bacteria in the human gut. Detecting ARGs in dairy products and milk is, therefore, crucial and could aid in the development of strategies to mitigate resistance dissemination through the food chain. **Objectives**: This study aimed to determine the presence of ARGs and assess the antibiotic susceptibility of LAB strains isolated from dairy products made from raw milk. **Methods**: Fifty-four LAB strains were isolated from 41 dairy samples and were tested for antimicrobial susceptibility using broth microdilution to determine Minimal Inhibitory Concentration (MIC). Moreover, the presence of resistance genes related to tetracyclines, beta-lactams, quinolones, and erythromycin was examined using six multiplex PCR assays. **Results**: *Lactobacillus* spp. and *Leuconostoc* spp. strains exhibited a high level of resistance to vancomycin (93–100%). Low-level resistance (4.2–20%) was observed in *Lactococcus* spp. and *Lactobacillus* spp. strains against tetracycline. Additionally, *Lactococcus* spp. strains showed resistance to trimethoprim/sulfamethoxazole, erythromycin, and clindamycin. Twenty-two out of 54 LAB strains (40.7%) carried at least one antibiotic resistance gene, and five of these were multidrug-resistant. Genes associated with acquired resistance to tetracycline were commonly detected, with *tet*K being the most frequent determinant. **Conclusions**: This study demonstrated that LABs in dairy products can act as reservoirs for ARGs, potentially contributing to the horizontal transfer of resistance within microbial communities in food and consumers. These findings highlight the need for the ongoing surveillance of antibiotic resistance in LAB and the implementation of control measures to minimize the dissemination of resistance through dairy products.

## 1. Introduction

Antibiotic resistance is a growing global concern, impacting not only human and animal health but also the safety and quality of food products [1]. Dairy products, in particular, provide a unique environment where microorganisms, especially Lactic Acid Bacteria (LAB), play a pivotal role in enhancing shelf-life, improving nutritional properties, and contributing to the distinctive organoleptic and structural qualities of products. For these reasons, LAB are intentionally added as starter cultures during production [2,3]. Among widely consumed dairy products, cheeses are particularly dependent on LAB activity, whether through natural cultures, selected starters, or as part of the adventitious microflora contaminating the raw material and processing environments. Conseque I checked ntly, cheese varieties on the market differ based on environmental conditions, microbial composition, and production technologies [4]. Dairy products made from raw milk with natural cultures typically have a more complex and heterogeneous lactic microflora than those made from pasteurized milk, where LAB primarily originate from selected starters [5]. The LAB community in these products includes mesophilic and thermophilic species with homo- and heterofermentative metabolism, primarily from genera, such as *Streptococcus*, *Lactococcus*, *Lactobacillus*, *Pediococcus*, *Leuconostoc*, and *Weissella* [6]. The type and number of LAB present vary depending on the dairy product and within the same type of product, such as from one cheese to another [7]. LAB are generally regarded as beneficial due to their probiotic properties and long history of safe use in food [8]. However, they can acquire or spread antibiotic resistance genes (ARGs) through horizontal gene transfer (HGT), such as transformation, transduction, and mobile genetic elements, like integrons, from other bacteria in the environment, livestock, or during food processing [9]. Through biofilms, dense microbial communities in dairy processing plants or on milking equipment facilitate close interactions between bacteria, increasing the likelihood of genetic exchange and this has been shown to occur both by conjugation and transformation [10]. Moreover, the presence of integrons in biofilms on dairy processing equipment may enhance resistance gene transfer among LAB and other microbes [10]. HGT via integrons may also allow commensal bacteria in the animal microbiota gut to acquire resistance genes from LAB, increasing the risk of antibiotic treatment failures [11]. Transfer of ARGs from live bacteria to other bacteria in the foodstuff or in the gut may also occur by conjugation or by transformation and transduction, although to a lesser extent [9]. Food processing and/or preservation techniques can kill or inactivate bacteria. Those dead bacterial cells may remain intact or may be lysed due to damage to the cell wall. As a result, bacterial DNA, including any ARGs present, are released into the environment and can theoretically be transferred by transformation although this process occurs at a low frequency [9]. Some studies also suggest that increasing the use of sublethal, rather than lethal, food preservation systems may contribute to the development and spread of antimicrobial resistance in pathogens through the conjugation of plasmids containing ARGs [12]. This poses a significant challenge, as resistant strains may not only survive antimicrobial treatments but also act as reservoirs for ARGs, transferring resistance via plasmids and transposons [13]. Plasmids are common in *Lactococcus*, *Pediococcus*, *Leuconostoc*, and *Streptococcus* and are present in certain species of *Lactobacillus* and *Streptococcus thermophilus* strains. Conjugative transposons have been described in *Lactococcus* and *Streptococcus*. Horizontal gene transfer involving *tet* genes between pathogenic (*Listeria monocytogenes*), potentially pathogenic (*Enterococcus faecalis*), and starter bacteria has been observed in dairy products [14]. To ensure consumer safety, it is essential that LAB used as starters or present in high quantities in dairy products do not harbor transmissible antibiotic resistance genes. While antibiotic-resistant LAB in dairy products may not pose an immediate health threat due to their typically non-pathogenic nature, their ability to transfer resistance genes to pathogenic microorganisms in food or the human gastrointestinal tract remains a serious concern [15,16,17]. This could lead to infections that are difficult to treat with conventional antibiotics. Moreover, the use of antibiotics in agriculture and food production has contributed to the rise of multidrug-resistant bacteria, which can spread through the food chain [18]. Dairy products, particularly those made from raw or minimally processed milk, may act as a vector for these resistant microorganisms. Therefore, monitoring LAB in widely consumed foods is crucial for assessing their potential as ARG reservoirs. This study aimed to assess the antibiotic susceptibility of LAB strains isolated from raw milk and its derived dairy products and to detect the presence of resistance genes to tetracyclines, beta-lactams, quinolones, and erythromycin.

## 2. Results

### 2.1. Strain Isolation and Identification

A total of 361 colonies were isolated from 41 dairy samples and identified using the MALDI-TOF system. The species identified are summarized in Supplementary Materials Table S1. The most represented genera were *Lactococcus* (49%, *n* = 177), *Streptococcus* (11%, *n* = 41), *Lactobacillus* (10%, *n* = 36), *Enterococcus* (9%, *n* = 31), and *Leuconostoc* (7%, *n* = 26). For subsequent analyses, 54 strains were selected: 24 *Lactococcus* spp., 15 *Lactobacillus* spp., eight *Leuconostoc* spp., and seven *Streptococcus* spp.

### 2.2. In Vitro Antimicrobial Susceptibility Testing

The 54 LAB strains were tested for resistance to 23 antibiotics (Supplementary Materials Table S2). In summary, 93.0% (*n* = 14) of the *Lactobacillus* spp. strains were resistant to vancomycin, 20.0% (*n* = 3) to tetracycline, and no resistance was observed to the other antibiotics tested (Table 1). Among *Lactococcus* spp. strains, 21% (*n* = 5) were resistant to trimethoprim/sulfamethoxazole, 12.5% (*n* = 3) to erythromycin, and 4.2% (*n* = 1) to both tetracycline and clindamycin (Table 2). *Streptococcus* spp. showed no resistance (Table 3). In addition, the resistance to vancomycin observed in the *Lactobacillus* (93%, *n* = 14) and *Leuconostoc* (100%, *n* = 8) genera is intrinsic (Table 4). No differences in antibiotic resistance were observed between strains within the same genus (Wilcoxon rank-sum test, *p* > 0.05).

### 2.3. PCR Analysis

Specific ARGs were searched by six multiplex PCR on 54 LAB strains. Twenty-two of these LAB (40.7%) showed at least one antibiotic resistance gene, and among these, 4/22 and 1/22 contained two and three resistance genes, respectively (Table 5). The genes contributing to acquired resistance to tetracycline were the most frequently observed (72.7%, *n* = 16), followed by those for beta-lactams (22.7%, *n* = 5), erythromycin (22.7%, *n* = 5), and quinolones (4.5%, *n* = 1). In tetracycline-resistant LAB strains, the most frequent *tet* determinant was *tet*K (62.5%, *n* = 10) followed by *tet*M (18.7%, *n* = 3), *tet*S (12.5%, *n* = 2), *tet*B, and *tet*L with the same frequency (6.3%, *n* = 1), while no strain possessed *tet*A and *tet*O. In erythromycin-positive LAB, the most frequent determinant was *erm*B (80%, *n* = 4), while in beta-lactams, it was *bla*TEM (60%, *n* = 3). The only determinant found in quinolone-positive LAB was *qnr*S. In LAB, the genera that most frequently showed resistance genes were those belonging to *Leuconostoc* spp. (62.5% *n* = 5) followed by *Lactococcus* spp. (41.7%, *n* = 10), *Lactobacillus* spp. (33.3%, *n* = 5), and *Streptococcus* spp. (28.6%, *n* = 2) (Table 5). Moreover, only the genera *Lactococcus* and *Streptococcus* showed the presence of multiple resistance genes (Table 2 and Table 3).

### 2.4. Agreement Between Antimicrobial Susceptibility Testing and Resistance Genotyping

The concordance analysis between antimicrobial resistance phenotype and genotype demonstrated variable agreement levels between the methods, depending on the specific antibiotic evaluated. For erythromycin, the results obtained through both methods indicate a substantial concordance (κ = 0.88; 95% CI = 0.64–1.0). In contrast, for the remaining resistance investigated, the concordance between the phenotype and genotype was minimal, as evidenced for tetracyclines (κ = 0.09; 95% CI = −0.14–0.32) or almost absent (κ = 0), as observed for betalactams (AMP, PEN) and quinolones (LEVO, MXF). For the latter antibiotics, the percentage of agreement between the two methods ranged from a minimum of 70.4% (95% CI = 57.8–83.0) to a maximum of 98.2% (95% CI = 94.4–100)

## 3. Discussion

The EU food contamination with veterinary drugs, including antibiotics, is under the strict control since the maximum residue levels in animal-derived food for human consumption are established in the Commission Regulation EU 37/2010 and monitored [22]. To assure the manufacturing of high-quality dairy products, operators are demanding high-quality antibiotic-free raw milk [23]; for this purpose, microbial inhibitory substances are tested to assure that the raw milk entering the food chain is free of antibiotic contamination. The use and misuse of antibiotics in the dairy industry and other food-producing sectors, such as in the treatment of mastitis [24,25,26], have contributed to the selection of resistant bacteria, making raw milk and dairy products potential reservoirs for antimicrobial-resistant bacteria or those carrying ARGs [25].

It has been well established that bacteria from the food chain, including dairy products, can facilitate the transfer of ARGs to intestinal bacteria through HGT events [27,28,29,30]. Specifically, both in vitro and in vivo studies have demonstrated the transfer of resistance genes from *Lactobacillus* spp. strains to Gram-positive bacteria, such as *Enterococcus faecalis* [31,32]. Given that LAB are a fundamental component of the dairy microbiota and play a crucial role in fermentation processes and sensory enhancement, their presence as reservoirs for ARGs poses a risk for the acquisition and dissemination of resistance, ultimately threatening consumer health [16,17,18]. Due to their beneficial or commensal nature, LAB has received limited scientific attention regarding antibiotic resistance. To address these concerns, this study investigated the antimicrobial resistance profiles of LAB strains isolated from raw milk and its derived dairy products.

The LAB group encompasses numerous highly heterogeneous bacterial species with distinct growth characteristics. Based on bibliographic data, two different growth media were selected, each incubated at two distinct temperatures, to maximize the microbial diversity of the strains included in the study [33,34,35]. The most prevalent genera identified in our study included *Lactococcus*, *Streptococcus*, *Lactobacillus,* and *Leuconostoc*, consistent with findings reported in other studies [33,36,37].

Since LABs are not considered pathogenic, official breakpoint values for antibiotic resistance are limited to only a few antibiotics in CLSI [19] and EUCAST guidelines [21]. In 2018, EFSA published the Guidance on the Characterization of Microorganisms Used as Feed Additives or Production Organisms [20], establishing breakpoints for certain LAB species [20]. No significant differences in phenotypic or genotypic antibiotic resistance were observed among the various dairy products, likely due to the limited sample size. Moreover, differences between bovine and caprine products were not assessed for the same reason. However, these comparisons were beyond the primary scope of this study. Susceptibility testing of LAB determined by MIC indicated that 10 strains were resistant to at least one antibiotic and that the highest levels of resistance were against tetracycline, trimethoprim/sulfamethoxazole, and erythromycin. The results obtained in this study showed that 13% (*n* = 3) of the strains *Lactobacillus* spp. were resistant to tetracyclines, which is the most extensively studied antibiotic class for this genus [31,32]. The majority of *Lactobacillus* spp. strains (93.3%, *n* = 14) exhibited resistance to vancomycin, in agreement with previous studies [38,39]: this resistance is generally considered an intrinsic characteristic of *Lactobacillus* spp., even if occasional cases of susceptibility have been reported [40,41]. All *Lactobacillus* spp. strains were found to be sensitive to the antibiotics daptomycin, linezolid, erythromycin, ampicillin, penicillin, clindamycin, chloramphenicol, gentamicin, and streptomycin. This finding contrasts with the results of Toomey et al. (2010), who observed a higher prevalence of erythromycin-resistant strains [40].

*Lactococcus* spp. strains were found to be resistant to trimethoprim-sulfamethoxazole (21%, *n* = 5), erythromycin (12.5%, *n* = 4), clindamycin (8.3% *n* = 2), and tetracycline (4.2% *n* = 1) as reported previously [42]. These antimicrobials are considered important for use in human medicine. According to the World Health Organization (WHO) classification, erythromycin belongs to the “watch group” [43,44]. This group includes classes of antibiotics that have a higher resistance potential and includes most of the top priority agents among the critically important antimicrobials for human medicine and/or antibiotics that are at a relatively high risk of bacterial resistance selection. Clindamycin, tetracycline, and trimethoprim-sulfamethoxazole are instead considered highly important antimicrobials and belong to the “access group”; this group shows a lower resistance potential than antibiotics in the other groups [43,44].

*Leuconostoc* spp. strains were found to be susceptible to ampicillin, chloramphenicol, erythromycin, gentamicin, penicillin, and tetracycline, in contrast to the findings reported previously by Flórez et al. (2005), which indicate resistance to tetracycline, chloramphenicol, and erythromycin [45]. All *Leuconostoc* spp. strains displayed phenotypic resistance to vancomycin, which is consistent with the existing literature [45,46,47,48].

Finally, *Streptococcus* spp. strains were phenotypically sensitive to all the antibiotics tested, which is in contrast to the findings reported by Flórez et al. (2017) that observed resistance to tetracycline, erythromycin, and clindamycin, streptomycin and neomycin [49].

Regarding the presence of resistance genes, in this study, we observed that 40.7% (*n* = 22) of the LAB strains carried at least one ARG, with a minority harboring multiple genes. The most frequently detected ARGs were tetracycline resistance genes, followed by beta-lactams, erythromycin, and quinolones genes. The genera most frequently harboring resistance genes were *Leuconostoc* spp. (62.5%, *n* = 5), followed by *Lactococcus* spp. (41.7%, *n* = 10), *Lactobacillus* spp. (33.3%, *n* = 5), and *Streptococcus* spp. (28.6%, *n* = 2). These findings demonstrate that LAB can serve as a reservoir of antibiotic resistance genes, as previously observed in other studies [38,49,50].

The presence of antibiotic resistance genes and their associated mechanisms has been extensively studied, with numerous reviews available in the literature [51,52,53]. Notably, it is emphasized that a silent gene does not prevent horizontal gene transfer and may become reactivated over time, potentially leading to phenotypic resistance [54]. This underscores the importance of studying antibiotic resistance from both phenotypic and genotypic perspectives, even in bacterial species not strictly associated with pathogenicity. The data obtained in this study revealed that phenotypic resistance did not always correlate with the presence of resistance genes. This phenomenon was observed across multiple bacterial species and antibiotic classes. Two strains of *Lactococcus garvieae* (ID 13 and 22) and one strain of *L. lactis* (ID 24), positive for the presence of the *erm*B gene, were found to be resistant to erythromycin, confirming the correlation between the resistance phenotype and the genotypic profile as observed by de Oliveira et al. (2022) [55]. Similarly, one strain of *Lactococcus lactis* (ID 2) showed resistance to tetracycline and tested positive for the presence of the *tet*S gene through PCR analysis. This result is consistent with CLSI [19], which states that tetracycline resistance observed in *L. lactis* and *L. garvieae* is attributed to the presence of the *tet*M or *tet*S genes. The tetracycline resistance observed in *Lactobacillus* spp. was confirmed by a strain of *Lactobacillus plantarum* (ID 11), which tested positive for the *tet*K resistance gene. However, one strain of *Lactobacillus delbrueckii* (ID 1) carried the *tet*M gene but remained phenotypically sensitive. A similar discrepancy has been previously reported in other studies [38]. In contrast, *Lactobacillus paracasei* (ID 7) and *Lactobacillus brevis* (ID 9) were negative for tetracycline resistance genes but were phenotypically resistant. This suggests that resistance may be associated with different genetic mechanisms that were not investigated in this study.

The low Cohen’s Kappa values for tetracyclines, beta-lactams, and quinolones suggest that the observed agreement may be potentially influenced by the unbalanced distribution of resistant and susceptible strains. These data suggest that while PCR is a cost-effective and easily applicable method, it may have limited predictive value for phenotypic resistance [56,57,58,59]. Therefore, further studies employing whole-genome sequencing (WGS) are necessary to gain deeper insights into the genetic mechanisms underlying resistance and their correlation with phenotypic expression [60,61,62].

Comparing our study with the existing literature reveals significant differences in reported resistance levels, which may stem from variations in methodology, geographic factors, and sample sources. Studies employing molecular methods often identify resistance genes in phenotypically susceptible strains, potentially leading to higher rates of resistance [63,64]. In particular, methods, such as WGS, may be further useful in studying mechanisms of HGT [65,66]. Geographic disparities in antibiotic use within dairy farming can further influence resistance patterns [67,68,69]. Additionally, variations in sample sources, such as raw versus pasteurized milk or different dairy products, may affect microbial composition and selective pressures, contributing to discrepancies in resistance findings [70,71,72]. For these reasons, conducting a comparative analysis with global trends is challenging and would require more focused and in-depth studies in the literature.

The generally low resistance levels observed in this study are encouraging from a public health perspective, as they suggest limited antibiotic pressure in the dairy production chain. This could be attributed to good farming and food production practices, including responsible antibiotic use in livestock, strict hygiene measures, and regulatory controls [73]. However, the results obtained here show that the LAB role in the food industry increases the risk of transferring resistance genes to other bacteria, including pathogenic ones [42,50]. Therefore, improving the knowledge of these strains is essential to better understand their potential as reservoirs of antibiotic resistance genes and to implement appropriate monitoring strategies to mitigate the risk of resistance dissemination through the food chain.

## 4. Materials and Methods

### 4.1. Dairy Samples

**Figure 1:**
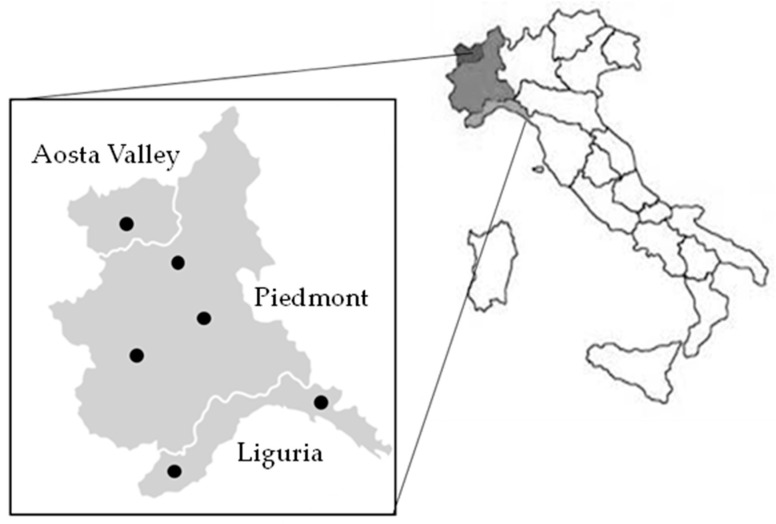
Map of sampling sites. The black circles indicate the collection points.

A total of 41 dairy products, including 16 raw milks, 4 curds, and 21 cheeses, were sampled at various stages of ripening from artisanal producers in three regions of Northwest Italy (Piedmont, Liguria and Aosta Valley); Figure 1. Emphasis was placed on raw milk products made without industrial starters to maximize microbial diversity. Sampling was conducted on a voluntary basis, with the number of samples depending on the dairy plant’s availability.

### 4.2. Strain Isolation and Identification

Solid samples (10 g) and liquid samples (10 mL) were each diluted with 90 mL of buffered peptone water (BPW, Microbiol, Cagliari, Italy). From this initial 10⁻¹ dilution, 1:10 serial dilutions were prepared up to 10^−7^ by transferring 1 mL of the starting solution into 9 mL of peptonated water (Microbiol, Cagliari, Italy). Subsequently, 1 mL of each dilution was placed in sterile Petri dishes (Biosigma, Venice, Italy) using a double-layer inclusion method. Two specific growth media for lactic acid bacteria were selected: De Man, Rogosa and Share (MRS, Microbiol, Cagliari, Italy) and M17 (Microbiol, Cagliari, Italy), which promote the growth of lactobacilli and lactococci, respectively. Two plates were prepared for each medium: one incubated at 37 °C for 48 h and the other at 30 °C for 72 h to ensure optimal growth conditions for diverse bacterial species. A variable number of colonies (ranging from 1 to 8), depending on concentration, were randomly selected from MRS and M17 plates, transferred onto blood agar, and incubated for 24 h under microaerophilic conditions. Identification of bacterial colonies was performed using a MALDI-TOF (Matrix-Assisted Laser Desorption/Ionization—Time of Flight) instrument (BRUKER, Billerica, MA, USA), following the supplier’s instructions (https://www.bruker.com/en/products-and-solutions/microbiology-and-diagnostics/microbial-identification/maldi-biotyper-sirius-system.html, accessed on 24 March 2025). The Bacterial Test Standard (BTS) provided by the company was used as a quality control. Among the identified strains, those belonging to the most represented genera were then selected for subsequent analyses, excluding those that may include possibly pathogenic species as *Enterococcus* spp. Strains were stored in CRYOBANK™ tubes (Copan, Brescia, Italy) and contained cryogenic preserving medium at −20 °C.

### 4.3. In Vitro Antimicrobial Susceptibility Testing

Antibiotic susceptibility was assessed using the broth microdilution method to determine the minimum inhibitory concentration (MIC). Sensititre™ GPALL1F and EULACBI1 (Thermo Fisher Scientific, Waltham, MA, USA) plates were employed, following the manufacturer’s instructions. American Type Culture Collection certified Enterococcus faecalis strain (ATCC 29212) was used as a control. In particular, *Lactobacillus* spp., *Lactococcus* spp., *Streptococcus* spp., and *Leuconostoc* spp. were tested using GPALL1F for the following antibiotics: ampicillin (AMP), 0.12–8 μg/mL; cefoxitin (FOX), 6 μg/mL; ciprofloxacin (CIP), 1–2 μg/mL; clindamycin (CLI), 0.5–2 μg/mL; chloramphenicol (CHL), 2–16 μg/mL; daptomycin (DAP), 0.5–4 μg/mL; erythromycin (ERY), 0.25–4 μg/mL; gentamicin (GEN), 2–16 μg/mL; levofloxacin (LEVO), 0.25–4 μg/mL; linezolid (LZD), 1–8 μg/mL; moxifloxacin (MXF), 0.25–4 μg/mL; nitrofurantoin (NIT), 32–64 μg/mL; oxacillin + 2% NaCl (OXA+), 0.25–4 μg/mL; penicillin (PEN), 0.06–8 μg/mL; quinupristin/dalfopristin (SYN), 0.5–4 μg/mL; rifampicin (RIF), 0.5–4 μg/mL; streptomycin (STR), 1000 μg/mL; tetracycline (TET), 2–16 μg/mL; tigecycline (TGC), 0.03–0.5 μg/mL; trimethoprim/sulfamethoxazole (STX), 0.5/9.5–4/76 μg/mL; vancomycin (VAN), 0.25–32 μg/mL. Additionally, *Lactobacillus* spp. and *Lactococcus* spp. were analyzed using EULACBI1 for the following antibiotics: clindamycin (CLI), 0.03–16 μg/mL; chloramphenicol (CHL), 0.12–64 μg/mL; erythromycin (ERY), 0.015–8 μg/mL; gentamicin (GEN), 0.5–256 μg/mL; kanamycin (KAN), 2–1024 μg/mL; neomycin (NEO), 0.12–64 μg/mL; streptomycin (STR), 0.5–256 μg/mL; tetracycline (TET), 0.12–64 μg/mL. The growth conditions recommended by CLSI [19] were applied: Cation-Adjusted Mueller Hinton Broth (CAMHB) supplemented with 2–5% lysed horse blood (LHB) (Thermo Fisher Scientific, Waltham, MA, USA) was used, and the plates were incubated for 24 h at 35 °C. The results were then compared to the thresholds established by CLSI M45 (3rd Edition), EUCAST 14 (2024), or EFSA (2018) [19,20,21]. Given the high variability of species included in the study, the breakpoints were assessed and reported according to the bacterial genera.

### 4.4. PCR Analysis

The selected LAB strains were screened for the presence of resistance genes related to tetracyclines, beta-lactams, quinolones, and erythromycin by PCR assays. These antibiotics or classes of antibiotics were chosen because they are used in both human and animal medicine, are considered to be of critical or highly important importance to human medicine, and the resistance genes associated with these classes are often transferable between bacteria [43,44].

For DNA extraction, a bacterial colony was resuspended in 500 μL of sterile Ultra-Pure water (Sigma-Aldrich, St. Louis, MO, USA) and heat-treated for 10 min at 99 °C with Thermomixer comfort (Eppendorf, Milan, Italy). The samples were then centrifuged at 10,000 g for 10 min with Microcentrifuge 5417R (Eppendorf, Milan, Italy). The extracted DNA was stored at −20 °C until amplification.

For DNA amplification, CFX96 Touch Real-Time PCR Detection System (Biorad, Hercules, CA, USA) was used. Six sets of multiplex PCR assays were selected for analysis based on available bibliographic sources: three multiplex PCRs to amplify seven tetracycline resistance genes, which represent the two main mechanisms of resistance to tetracyclines, the ribosomal one and the efflux one: (I) *tet*B; (II) *tet*A; (III) *tet*K, *tet*L, *tet*M, *tet*O, *tet*S [74]; one multiplex PCR to detect the presence of two *bla* genes responsible for beta-lactam antibiotic resistance: (IV) *bla*CTX-M and *bla*TEM [75]. One multiplex PCR to identify three *qnr* genes associated with quinolone resistance: (V) *qnr*A, *qnr*B, *qnr*S [76]. One multiplex PCR to detect three erm genes responsible for erythromycin resistance: (VI) *erm*A, *erm*B, *erm*C [77].

The PCR multiplex reactions were prepared by mixing, in a final volume of 25 μL, 12.5 μL of DreamTaq Hot Start PCR Master Mix (2x) (Thermo Fisher Scientific, Waltham, MA, USA), and 1 μL of each primer pair (Metabion, Planegg, Germany) to a final concentration of 10 µM, and 5 μL of DNA. The amplification conditions were as follows: (1) Initial denaturation, 95 °C for 3 min; (2) Denaturation, 95 °C for 30 s; (3) Annealing, specific temperatures for each primer set (as listed in Table 6); (4) Elongation, 72 °C for 1 min; and (5) Final elongation, 72 °C for 10 min. Steps 2, 3, and 4 were repeated for 35 cycles.

Amplicons were visualized using capillary electrophoresis on a QIAxcel Advanced System instrument (QIAGEN, Hilden, Germany) following the supplier’s instructions [78]. The lengths of the amplified products, as listed in Table 6, were compared to those of a known molecular weight marker (Bio-Rad, Hercules, CA, USA).

### 4.5. Statistical Analysis

Data on bacterial isolation, species identification, and antimicrobial resistance profiles (phenotypes and genotypes) were managed and analyzed using Stata 17 [79]. Descriptive statistics were employed to calculate the frequency of antibiotic resistance, expressed as percentages for each LAB genus and the tested antimicrobials.

To assess differences in antibiotic resistance, the non-parametric Wilcoxon rank-sum test (Mann–Whitney U test) was performed. Comparisons were conducted within each LAB genus, using the most prevalent bacterial species as the reference group, while less frequent species were grouped together as the comparison group. Additionally, the Cohen’s Kappa test was used to evaluate the agreement between the results obtained from antimicrobial susceptibility testing and resistance genotyping. A statistical significance level of 5% (*p* < 0.05) was set for all analyses.

## 5. Conclusions

LAB represents a fundamental component of the microbiota in dairy products, playing a crucial role in fermentation processes and enhancing sensory characteristics. However, despite their generally recognized safety, these bacteria can act as reservoirs for antibiotic resistance genes, posing a potential risk for horizontal gene transfer within microbial communities. Specifically, the predominant phenotypic resistances identified in this study were against tetracycline, trimethoprim/sulfamethoxazole, and erythromycin.

This study highlights the importance of investigating antibiotic resistance in lactic acid bacteria from both phenotypic and genotypic perspectives. While most strains were sensitive to key antibiotics, the presence of resistance genes in phenotypically sensitive strains underscores the potential for gene reactivation and horizontal transfer, emphasizing the need for continued monitoring even in non-pathogenic bacterial species used in dairy production.

Certain methodological constraints must be acknowledged, including the sample size and the use of PCR-based detection, which may not comprehensively capture all resistance determinants or their expression levels. Future investigations should incorporate whole-genome sequencing (WGS) to provide a more comprehensive characterization of resistance mechanisms and their potential for dissemination. Expanding the scope to include a wider range of dairy products and production environments could further elucidate the factors influencing antimicrobial resistance in LAB.

## Figures and Tables

**Table 1:** Antimicrobial susceptibility and antibiotic resistance genes of *Lactobacillus* spp. strains. Susceptible (S), intermediate (**I**), and resistant (**R**).

ID	LAB Strain	Sample Type	AMP ^1^	CHL ^2^	CLI ^1^	DAP^1^	ERY ^1^	GEN ^2^	KAN ^2^	LZD ^1^	PEN ^1^	STR ^2^	TET ^2^	VAN ^1^	Antibiotic Resistance Gene
1	*L. delbrueckii*	Cow milk	S	S	S	S	S	S	S	S	S	S	S	S	*tet*M
2	*L. fermentum*	Cow milk	S	S	S	S	S	S	S	S	S	S	S	**R**	-
3	*L. curvatus*	Goat cheese	S	S	S	S	S	S	S	S	S	S	S	**R**	-
4	*L. paracasei*	Cow cheese	S	S	S	S	S	S	S	S	S	S	S	**R**	-
5	*L. paracasei*	Goat cheese	S	S	S	S	S	S	S	S	S	S	S	**R**	-
6	*L. plantarum*	Cow cheese	S	S	S	S	S	S	S	S	S	/	S	**R**	-
7	*L. paracasei*	Cow cheese	S	S	S	S	S	S	S	S	S	S	**R**	**R**	-
8	*L. plantarum*	Cow cheese	S	S	S	S	S	S	S	S	S	/	S	**R**	-
9	*L. brevis*	Goat cheese	S	S	S	S	S	S	S	S	S	S	**R**	**R**	-
10	*L. paracasei*	Cow milk	S	S	S	S	S	S	S	S	S	S	S	**R**	*bla*TEM
11	*L. plantarum*	Cow cheese	S	S	S	S	S	S	S	S	S	/	**R**	**R**	*tet*K
12	*L. paracasei*	Cow cheese	S	S	S	S	S	S	S	S	S	S	S	**R**	-
13	*L. paracasei*	Cow cheese	S	S	S	S	S	S	S	S	S	S	S	**R**	-
14	*L. paracasei*	Cow cheese	S	**I**	S	S	S	S	S	S	S	S	S	**R**	*bla*TEM
15	*L. curvatus*	Cow cheese	S	S	S	S	S	S	S	S	S	S	S	**R**	*bla*TEM
Resistance rate (%)													20.0	93.3	

^1^ breakpoints provided by Clinical and Laboratory Standards Institute (CLSI) [19]; ^2^ breakpoints provided by European Food Safety Authority (EFSA) [20]. AMP, Ampicillin; CHL, Chloramphenicol; CLI, Clindamycin; DAP, Daptomycin; ERY, Erythromycin; GEN, Gentamycin; KAN, Kanamycin; LZD, Linezolid; PEN, Penicillins; STR, Streptomycin; TET, Tetracycline; VAN, Vancomycin. Not applicable (/); not detected (-).

**Table 2:** Antimicrobial susceptibility and antibiotic resistance genes of *Lactococcus* spp. strains. Susceptible (S) and resistant (**R**).

ID	LAB Strain	Sample Type	AMP ^1^	CHL ^2^	CLI ^1^	ERY ^1^	GEN ^2^	KAN ^2^	LEVO ^1^	PEN ^1^	STR ^2^	SXT ^1^	TET ^1^	VAN ^1^	Antibiotic Resistance Genes
1	*L. lactis*	Cow cheese	S	S	S	S	S	S	S	S	S	**R**	S	S	-
2	*L. lactis*	Cow cheese	S	S	S	S	S	S	S	S	S	**R**	**R**	S	*tet*S
3	*L. lactis*	Cow curd	S	S	S	S	S	S	S	S	S	**R**	S	S	*tet*K
4	*L. lactis*	Cow milk	S	S	S	S	S	S	S	S	S	S	S	S	-
5	*L. lactis*	Cow curd	S	S	S	S	S	S	S	S	S	S	S	S	-
6	*L. lactis*	Cow cheese	S	S	S	S	S	S	S	S	S	S	S	S	-
7	*L. lactis*	Cow cheese	S	S	S	S	S	S	S	S	S	S	S	S	-
8	*L. lactis*	Goat cheese	S	S	S	S	S	S	S	S	S	S	S	S	-
9	*L. lactis*	Cow cheese	S	S	S	S	S	S	S	S	S	S	S	S	*tet*K
10	*L. lactis*	Goat cheese	S	S	S	S	S	S	S	S	S	S	S	S	*tet*K
11	*L. lactis*	Cow cheese	S	S	S	S	S	S	S	S	S	S	S	S	*tet*K
12	*L. lactis*	Cow cheese	S	S	S	S	S	S	S	S	S	S	S	S	*tet*M
13	*L. garvieae*	Cow cheese	S	/	**R**	**R**	/	/	S	S	/	**R**	S	S	*tet*B, *erm*B
14	*L. lactis*	Cow milk	S	S	S	S	S	S	S	S	S	S	S	S	-
15	*L. raffinolactis*	Cow milk	S	/	S	S	/	/	S	S	/	S	S	S	-
16	*L. lactis*	Cow curd	S	S	S	S	S	S	S	S	S	S	S	S	-
17	*L. lactis*	Cow milk	S	S	S	S	S	S	S	S	S	S	S	S	-
18	*L. lactis*	Cow curd	S	S	S	S	S	S	S	S	S	S	S	S	-
19	*L. lactis*	Cow cheese	S	S	S	S	S	S	S	S	S	S	S	S	-
20	*L. lactis*	Cow cheese	S	S	S	S	S	S	S	S	S	S	S	S	-
21	*L. lactis*	Cow cheese	S	S	S	S	S	S	S	S	S	S	S	S	-
22	*L. garvieae*	Cow cheese	S	/	S	**R**	/	/	S	S	/	**R**	S	S	*tet*K, *erm*B
23	*L. lactis*	Cow cheese	S	S	S	**R**	S	S	S	S	S	S	S	S	*tet*K, *erm*A
24	*L. lactis*	Cow cheese	S	S	**R**	**R**	S	S	S	S	S	S	S	S	*erm*B
Resistance rate (%)					8.3	12.5						21.0	4.2		

^1^ breakpoints provided by CLSI [19]; ^2^ breakpoints provided by EFSA [20]. AMP, Ampicillin; CHL, Chloramphenicol; CLI, Clindamycin; ERY, Erythromycin; GEN, Gentamycin; KAN, Kanamycin; LEVO, Levofloxacin; PEN, Penicillins; STR, Streptomycin; SXT, Trimethoprim/Sulfamethoxazole; TET, Tetracycline; VAN, Vancomycin. Not applicable (/); not detected (-).

**Table 3:** Antimicrobial susceptibility and antibiotic resistance genes of *Streptococcus* spp. strains. Susceptible (S).

ID	LAB Strain	Sample Type	AMP ^1^	CHL ^1^	CLI ^1^	DAP ^1^	ERY ^1^	GEN ^2^	LEVO ^1^	LZD ^1^	MXF ^2^	PEN ^1^	RIF ^2^	SYN ^1^	TET ^1^	VAN ^1^	Antibiotic Resistance Genes
1	*S. salivarius* spp. *thermophilus*	Cow curd	S	S	S	S	S	S	S	S	S	S	S	S	S	S	-
2	*S. salivarius* spp. *thermophilus*	Cow cheese	S	S	S	S	S	S	S	S	S	S	S	S	S	S	-
3	*S. salivarius* spp. *thermophilus*	Cow cheese	S	S	S	S	S	S	S	S	S	S	S	S	S	S	-
4	*S. equinus*	Cow milk	S	S	S	S	S	S	S	S	S	S	S	S	S	S	*bla*CTX-M*, erm*B
5	*S. salivarius* spp. *thermophilus*	Cow curd	S	S	S	S	S	S	S	S	S	S	S	S	S	S	*tet*K*, tet*L*, bla*CTX-M
6	*S. salivarius* spp. *thermophilus*	Cow cheese	S	S	S	S	S	S	S	S	S	S	S	S	S	S	-
7	*S. salivarius* spp. *thermophilus*	Cow cheese	S	S	S	S	S	S	S	S	S	S	S	S	S	S	-

^1^ breakpoints provided by CLSI [19]; ^2^ breakpoints provided by European Committee on Antimicrobial Susceptibility Testing (EUCAST) [21]. AMP, Ampicillin; CHL, Chloramphenicol; CLI, Clindamycin; DAP, Daptomycin; ERY, Erythromycin; GEN, Gentamycin; LEVO, Levofloxacin; LZD, Linezolid; MXF, Moxifloxacin; PEN, Penicillins; RIF, Rifampicin; SYN; Quinupristin/Dalfopristin; TET, Tetracycline; VAN, Vancomycin. Not detected (-).

**Table 4:** Antimicrobial susceptibility and antibiotic resistance genes of *Leuconostoc* spp. strains. Susceptible (S) and resistant (**R**).

ID	LAB Strain	Sample Type	AMP ^1^	CHL ^1^	ERY ^1^	GEN ^1^	PEN ^1^	TET ^1^	VAN ^1^	Antibiotic Resistance Genes
1	*L. mesenteroides*	Cow Cheese	S	S	S	S	S	S	**R**	*tet*S
2	*L. mesenteroides*	Goat cheese	S	S	S	S	S	S	**R**	-
3	*L. pseudomesenteroides*	Cow curd	S	S	S	S	S	S	**R**	*tet*K
4	*L. mesenteroides*	Cow curd	S	S	S	S	S	S	**R**	*qnr*S
5	*L. mesenteroides*	Cow curd	S	S	S	S	S	S	**R**	-
6	*L. mesenteroides*	Cow Cheese	S	S	S	S	S	S	**R**	*tet*K
7	*L. pseudomesenteroides*	Cow Cheese	S	S	S	S	S	S	**R**	*tet*M
8	*L. mesenteroides*	Cow Cheese	S	S	S	S	S	S	**R**	-
Resistance rate (%)									100.0	

^1^ breakpoints provided by CLSI [19]. AMP, Ampicillin; CHL, Chloramphenicol; ERY, Erythromycin; GEN, Gentamycin; PEN, Penicillins; TET, Tetracycline; VAN, Vancomycin. Not detected (-).

**Table 5:** Antibiotic resistance genes detected in the LAB isolated from different dairy products.

LAB Strains	Resistance Genes			
	Tetracycline	*β*-Lactamase	Erythromycin	Quinolone
*Lactococcus lactis* (*n =* 21)	5/22 *tet*K, 1/22 *tet*M, 1/22 *tet*S	*-*	1/22 *erm*A, 1/22 *erm*B	-
*Lactobacillus delbrueckii* (*n* = 1)	1/1 *tet*M	-	-	-
*Lactobacillus fermentum* (*n* = 1)	-	-	-	-
*Streptococcus salivarius* ssp *thermophilus* (*n* = 6)	1/6 *tet*K, 1/6 *tet*L	1/6 *bla*CTX-M	-	-
*Leuconostoc mesenteroides* (*n* = 6)	1/6 *tet*K, 1/6 *tet*S	-	-	1/6 *qnr*S
*Lactobacillus curvatus* (*n* = 2)	-	1/2 *bla*TEM	-	-
*Lactococcus garvieae (n* = 2)	1/2 *tet*B, 1/2 *tet*K	-	2/2 *erm*B	-
*Lactobacillus plantarum* (*n* = 3)	1/3 *tet*K	-	-	-
*Lactobacillus paracasei* (*n* = 3)	-	-	-	-
*Lactobacillus brevis (n* = 1)	-	-	-	-
*Streptococcus equinus (n* = 1)	-	1/1 *bla*CTX-M	1/1 *erm*B	-
*Lacticaseibacillus paracasei (n* = 4)	-	2/4 *bla*TEM	-	--
*Lactococcus raffinolactis (n* = 1*)*	-	-	-	
*Leuconostoc pseudomesenteroides (n* = 2)	1/2 *tet*M, 1/2 *tet*K	-	-	-

Not detected (-).

**Table 6:** Target genes, primer sequences, annealing temperature, and amplicon size related to the multiplex PCRs used in the study. All primers were sourced from the published literature [74,75,76,77].

Target Genes	Primer Sequence (5′–3′)	Annealing Temperature	Amplified Size (bp)	References
*tet*B	TTG GTT AGG GGC AAG TTT TG	55 °C	659	[74]
	GTA ATG GGC CAA TAA CAC CG			
*tet*A	GCT ACA TCC TGC TTG CCT TC	55 °C	210	[74]
	CAT AGA TCG CCG TGA AGA GG			
*tet*K	TCG ATA GGA ACA GCA GTA	55 °C	169	[74]
	CAG CAG ATC CTA CTC CTT			
*tet*L	TCGTTA GCGTGC TGTCAT TC	55 °C	267	[74]
	GTATCCCACCAATGTAGCCG			
*tet*M	GTGGACAAAGGT ACA ACGAG	55 °C	406	[74]
	CGGTAAAGTTCGTCA CACAC			
*tet*O	AACTTAGGCATTCTGGCTCAC	55 °C	515	[74]
	TCC CACTGTTCC ATATCGTCA			
*tet*S	CAT AGA CAA GCCGTT GACC	55 °C	667	[74]
	ATG TTT TTG GAACGC CAG AG			
*bla*CTX-M	ATG TGCAGYACCAGTAARGTKATGGC	62 °C	593	[75]
	TGG GTRAARTARGTSACCAGAAYCAGCGG			
*bla*TEM	CGCCGCATACACTATTCTCAGAATGA	62 °C	445	[75]
	ACGCTCACCGGCTCCAGATTTAT			
*qnr*A	ATTTCTCACGCCAGGATTTG	53 °C	516	[76]
	GATCGGCAAAGGTTAGGTCA			
*qnr*B	GATCGTGAAAGCCAGAAAGG	53 °C	469	[76]
	ACGATGCCTGGTAGTTGTCC			
*qnr*S	ACGACATTCGTCAACTGCAA	53 °C	417	[76]
	TAAATTGGCACCCTGTAGGC			
*erm*A	GTTCAAGAACAATCAATACAGAG GGATCAGGAAAAGGACATTTTAC	53 °C	421	[77]
*erm*B	CCGTTTACGAAATTGGAACAGGTAAAGGGC GAATCGAGACTTGAGTGTGC	53 °C	359	[77]
*erm*C	GCTAATATTGTTTAAATCGTCAATTCC GGATCAGGAAAAGGACATTTTAC	53 °C	572	[77]

## Data Availability

The original contributions presented in this study are included in the article/Supplementary Material. Further inquiries can be directed to the corresponding author.

## Supplementary Materials

The following supporting information can be downloaded at: https://www.mdpi.com/article/10.3390/antibiotics14040375/s1, Table S1: Identification of colonies isolated from the dairy products with MALDI TOF; Table S2: Minimum Inhibitory Concentration (MIC) values obtained for 54 LAB strains tested for their resistance to antibiotics.
