# Supplementary material for: Antibiotic Resistance in Lactic Acid Bacteria from Dairy Products in Northern Italy
Source: Antibiotics (Basel). 2025 Apr 3;14(4):375. doi: 10.3390/antibiotics14040375 (PMC12024235; doi:10.3390/antibiotics14040375)
Supplement: Supplementary file 1 [file antibiotics-14-00375-s001.zip › antibiotics-3504068-supplementary.pdf]

Table S1. Identification of colonies isolated from the dairy products with MALDI TOF

**Species**	**Number of isolates**
Carnobacterium maltaromaticum	1
Citrobacter braakii	2
Enterobacter bugandensis	4
Enterobacter cloacae	1
Enterobacter kobei	2
Enterobacter ludwigii	1
Enterococcus durans	4
Enterococcus faecalis	20
Enterococcus faecium	5
Enterococcus gilvus	2
Hafnia alvei	7
Klebsialla oxytoca	4
Kluyveromyces marxianus	1
Kocuria varians	4
Kurthia gibsonii	2
Lactobacillus paracasei	23
lactobacillus brevis	1
Lactobacillus curvatus	1
Lactobacillus delbrueckii	1
Lactobacillus fermentum	2
Lactobacillus plantarum	8
Lactococcus garvieae	39
Lactococcus lactis	136
Lactococcus raffinolactis	2
Lelliottia amnigena	1
Leuconostoc mesenteroides	22
Leuconostoc pseudomesenteroides	4
Pediococcus pentosaceus	3
Rahnella aquatilis	1
Raoutella ornithinolytica	2
Serratia liquefaciens	2
Serratia marcescens	7
Staphylococcus aureus	3
Staphylococcus epidermidis	2
Staphylococcus hominis	1
Streptococcus agalactiae	1
Streptococcus equinus	2
Streptococcus gallolyticus	2
Streptococcus parauberis	2
Streptococcus salivarius ssp *thermophilus*	33
Streptococcus uberis	1

Table S2. Minimum Inhibitory Concentration (MIC) values obtained for 54 LAB strains tested for their resistance to antibiotics.

ID	LAB strain	GPALL1F																					EULACBI1							
		**CHL**	**DAP**	**GEN**	**LZD**	**RIF**	**SXT**	**SYN**	**TET**	**ERY**	**OXA+**	**AMP**	**PEN**	**VAN**	**LEVO**	**TGC**	**MXF**	**CLI**	**STR**	**CIP**	**NIT**	**FOXS**	**GEN**	**KAN**	**STR**	**NEO**	**TET**	**ERY**	**CLI**	**CHL**
		**2-16 µg/mL**	**0.5-4 µg/mL**	**2-16 µg/mL**	**1-8 µg/mL**	**0.5-4 µg/mL**	**0.5/9.5-4/76 µg/mL**	**0.5-4 µg/mL**	**2-16 µg/mL**	**0.25-4 µg/mL**	**0.25-4 µg/mL**	**0.12-8 µg/mL**	**0.06-8 µg/mL**	**0.25-32 µg/mL**	**0.25-4 µg/mL**	**0.03-0.5 µg/mL**	**0.25-4 µg/mL**	**0.5-2 µg/mL**	**1000 µg/mL**	**1-2 µg/mL**	**32-64 µg/mL**	**6 µg/mL**	**0.5-256 µg/mL**	**2-1024 µg/mL**	**0.5-256 µg/mL**	**0.12-64 µg/mL**	**0.12-64 µg/mL**	**0.015-8 µg/mL**	**0.03-16 µg/mL**	**0.12-64 µg/mL**
1	Lactobacillus delbrueckii	≤2	≤0.5	≤2	≤1	≤0.5	≤0.5/9.5	≤0.5	≤2	≤0.25	≤0.25	0.25	≤0.06	≤0.25	2	≤0.03	≤0.25	≤0.5	≤1000	2	≤32	≤6	≤0.5	≤2	≤0.5	≤0.12	≤0.12	≤0.015	≤0.03	0.5
2	Lactobacillus fermentum	≤2	≤0.5	≤2	≤1	≤0.5	≤0.5/9.5	≤0.5	≤2	≤0.25	≤0.25	≤0.12	≤0.06	>32	2	≤0.03	≤0.25	≤0.5	≤1000	≤1	≤32	≤6	≤0.5	≤2	≤0.5	≤0.12	≤0.12	≤0.015	≤0.03	0.5
3	Lactobacillus curvatus	≤2	≤0.5	≤2	≤1	≤0.5	>4/76	≤0.5	≤2	≤0.25	2	0.5	0.5	>32	4	≤0.03	≤0.25	≤0.5	≤1000	>2	64	>6	≤0.5	4	16	1	1	≤0.015	≤0.03	1
4	Lactobacillus paracasei	≤2	≤0.5	≤2	≤1	≤0.5	≤0.5/9.5	≤0.5	≤2	≤0.25	≤0.25	1	1	>32	1	≤0.03	≤0.25	≤0.5	≤1000	≤1	≤32	>6	≤0.5	8	8	1	0.5	≤0.015	≤0.03	1
5	Lactobacillus paracasei	≤2	≤0.5	≤2	≤1	≤0.5	≤0.5/9.5	≤0.5	≤2	≤0.25	≤0.25	1	0.5	>32	1	≤0.03	≤0.25	≤0.5	≤1000	≤1	≤32	>6	1	16	8	1	0.5	≤0.015	≤0.03	1
6	Lactobacillus plantarum	≤2	≤0.5	≤2	≤1	≤0.5	≤0.5/9.5	1	≤2	≤0.25	2	1	0.5	>32	1	≤0.03	≤0.25	≤0.5	≤1000	≤1	≤32	>6	≤0.5	16	8	1	0.5	≤0.015	≤0.03	1
7	Lactobacillus paracasei	≤2	≤0.5	≤2	≤1	4	≤0.5/9.5	1	16	≤0.25	≤0.25	1	4	>32	4	0.5	1	≤0.5	≤1000	>2	≤32	>6	≤0.5	≤2	4	≤0.12	4	≤0.015	0.12	1
8	Lactobacillus plantarum	≤2	≤0.5	≤2	2	2	≤0.5/9.5	≤0.5	8	≤0.25	≤0.25	0.5	1	>32	4	0.5	1	≤0.5	≤1000	>2	≤32	>6	≤0.5	≤2	2	≤0.12	8	≤0.015	0.12	1
9	Lactobacillus brevis	≤2	≤0.5	≤2	≤1	≤0.5	≤0.5/9.5	1	16	≤0.25	≤0.25	0.5	1	>32	4	0.06	0.5	≤0.5	≤1000	>2	≤32	>6	≤0.5	≤2	1	≤0.12	4	≤0.015	0.12	0.5
10	Lacticaseibacillus paracasei	≤2	≤0.5	≤2	≤1	≤0.5	≤0.5/9.5	≤0.5	≤2	≤0.25	≤0.25	1	0.5	>32	1	≤0.03	≤0.25	≤0.5	≤1000	≤1	≤32	>6	≤0.5	4	4	1	0.25	≤0.015	≤0.03	≤0.12
11	Lactobacillus plantarum	4	≤0.5	≤2	2	2	≤0.5/9.5	1	>16	≤0.25	4	1	2	>32	4	0.12	0.5	≤0.5	≤1000	>2	≤32	>6	≤0.5	≤2	1	≤0.12	32	≤0.015	0.12	1
12	Lacticaseibacillus paracasei	≤2	≤0.5	≤2	≤1	≤0.5	≤0.5/9.5	1	≤2	≤0.25	1	1	0.5	>32	1	0.12	≤0.25	≤0.5	≤1000	≤1	≤32	>6	≤0.5	8	4	2	0.5	≤0.015	≤0.03	0.5
13	Lacticaseibacillus paracasei	≤2	≤0.5	≤2	≤1	≤0.5	≤0.5/9.5	1	≤2	≤0.25	2	1	0.5	>32	1	0.12	≤0.25	≤0.5	≤1000	≤1	≤32	>6	≤0.5	16	8	2	0.5	≤0.015	≤0.03	1
14	Lacticaseibacillus paracasei	4	≤0.5	≤2	≤1	≤0.5	≤0.5/9.5	1	≤2	≤0.25	2	2	1	>32	1	≤0.03	≤0.25	≤0.5	≤1000	≤1	≤32	>6	≤0.5	4	4	0.5	1	≤0.015	≤0.03	2
15	Lactobacillus curvatus	≤2	≤0.5	≤2	≤1	≤0.5	≤0.5/9.5	≤0.5	≤2	≤0.25	1	1	0.5	>32	4	≤0.03	1	≤0.5	≤1000	>2	≤32	>6	≤0.5	4	8	0.5	1	0.03	≤0.03	2
1	Lactococcus lactis	8	≤0.5	≤2	2	>4	>4/76	2	<2	≤0.25	1	0.25	0.5	≤0.25	0.5	0.12	≤0.25	≤0.5	≤1000	2	64	>6	≤0.5	4	16	1	0.25	0.03	0.06	2
2	Lactococcus lactis	8	1	≤2	2	>4	>4/76	2	>16	≤0.25	1	0.25	0.5	≤0.25	≤0.25	≤0.03	≤0.25	≤0.5	≤1000	2	≤32	>6	≤0.5	≤2	8	1	64	≤0.015	≤0.03	≤0.12
3	Lactococcus lactis	8	≤0.5	≤2	4	>4	>4/76	2	<2	≤0.25	≤0.25	≤0.12	0.5	≤0.25	1	0.12	≤0.25	≤0.5	≤1000	>2	≤32	>6	≤0.5	≤2	1	≤0.12	≤0.12	≤0.015	≤0.03	0.25
4	Lactococcus lactis	4	≤0.5	≤2	2	>4	≤0.5/9.5	2	≤2	≤0.25	1	0.25	0.5	≤0.25	1	0.12	≤0.25	≤0.5	≤1000	>2	64	>6	≤0.5	≤2	4	0.5	0.25	0.03	0.06	1
5	Lactococcus lactis	4	≤0.5	≤2	2	>4	≤0.5/9.5	2	≤2	≤0.25	2	0.25	0.5	≤0.25	1	0.12	≤0.25	≤0.5	≤1000	>2	>64	>6	1	4	8	1	0.25	0.03	0.06	1
6	Lactococcus lactis	4	≤0.5	≤2	2	>4	≤0.5/9.5	2	≤2	≤0.25	2	0.25	0.5	≤0.25	1	0.12	≤0.25	≤0.5	≤1000	>2	>64	>6	≤0.5	4	16	1	0.25	0.03	0.06	1
7	Lactococcus lactis	4	≤0.5	≤2	2	>4	≤0.5/9.5	2	≤2	≤0.25	2	0.25	0.5	≤0.25	1	0.12	≤0.25	≤0.5	≤1000	>2	>64	>6	1	≤2	8	1	0.25	0.03	0.06	≤0.12
8	Lactococcus lactis	4	≤0.5	≤2	2	>4	≤0.5/9.5	1	≤2	≤0.25	0.5	0.25	0.5	≤0.25	1	0.06	≤0.25	≤0.5	≤1000	2	>64	>6	≤0.5	≤2	8	1	≤0.12	0.03	≤0.03	1
9	Lactococcus lactis	4	≤0.5	≤2	2	>4	≤0.5/9.5	1	≤2	≤0.25	1	0.25	0.25	≤0.25	1	0.06	≤0.25	≤0.5	≤1000	2	>64	>6	≤0.5	≤2	8	0.25	0.25	0.03	≤0.03	1
10	Lactococcus lactis	4	≤0.5	≤2	2	>4	≤0.5/9.5	1	≤2	≤0.25	1	0.25	0.25	≤0.25	0.5	0.06	≤0.25	≤0.5	≤1000	2	>64	>6	≤0.5	≤2	8	0.5	≤0.12	0.03	≤0.03	≤0.12
11	Lactococcus lactis	≤2	≤0.5	≤2	≤1	4	≤0.5/9.5	2	≤2	≤0.25	0.5	≤0.12	0.25	≤0.25	0.5	0.06	≤0.25	≤0.5	≤1000	≤1	≤32	>6	≤0.5	≤2	8	0.5	≤0.12	≤0.015	≤0.03	1
12	Lactococcus lactis	4	≤0.5	≤2	2	4	≤0.5/9.5	2	≤2	≤0.25	0.5	≤0.12	0.5	≤0.25	0.5	0.06	≤0.25	≤0.5	≤1000	≤1	≤32	>6	≤0.5	≤2	4	0.25	0.25	0.03	≤0.03	1
13	Lactococcus garvieae	>16	≤0.5	4	≤1	>4	4/76	>4	≤2	>4	>4	1	1	0.5	1	0.12	≤0.25	>2	≤1000	2	≤32	>6	≤0.5	16	128	16	0.25	>8	>16	32
14	Lactococcus lactis	≤2	≤0.5	≤2	≤1	>4	≤0.5/9.5	2	≤2	≤0.25	≤0.25	≤0.12	0.25	≤0.25	0.5	0.12	≤0.25	≤0.5	≤1000	2	≤32	>6	≤0.5	≤2	4	0.5	≤0.12	0.03	≤0.03	1
15	Lactococcus raffinolactis	≤2	≤0.5	≤2	≤1	≤0.5	≤0.5/9.5	≤0.5	≤2	≤0.25	≤0.25	≤0.12	0.25	0.5	0.5	≤0.03	≤0.25	≤0.5	≤1000	≤1	≤32	>6	≤0.5	4	16	2	≤0.12	≤0.015	≤0.03	1
16	Lactococcus lactis	≤2	≤0.5	≤2	≤1	4	≤0.5/9.5	2	<2	≤0.25	≤0.25	≤0.12	0.25	≤0.25	0.5	0.06	≤0.25	≤0.5	≤1000	2	≤32	>6	≤0.5	≤2	4	0.25	≤0.12	≤0.015	≤0.03	1
17	Lactococcus lactis	4	≤0.5	≤2	2	>4	≤0.5/9.5	2	<2	≤0.25	0.5	≤0.12	0.25	≤0.25	0.5	0.12	≤0.25	≤0.5	≤1000	≤1	≤32	>6	≤0.5	≤2	4	0.5	0.25	0.03	≤0.03	0.25
18	Lactococcus lactis	4	≤0.5	≤2	≤1	>4	≤0.5/9.5	≤0.5	<2	≤0.25	1	0.25	0.5	0.5	0.5	≤0.03	≤0.25	≤0.5	≤1000	2	≤32	>6	≤0.5	≤2	8	1	≤0.12	≤0.015	≤0.03	1
19	Lactococcus lactis	≤2	≤0.5	≤2	≤1	>4	≤0.5/9.5	2	<2	≤0.25	0.5	≤0.12	0.25	≤0.25	0.5	≤0.03	≤0.25	≤0.5	≤1000	≤1	≤32	>6	≤0.5	≤2	4	0.25	≤0.12	≤0.015	≤0.03	1
20	Lactococcus lactis	4	≤0.5	≤2	2	>4	≤0.5/9.5	2	<2	≤0.25	0.5	≤0.12	0.25	≤0.25	0.5	0.12	≤0.25	≤0.5	≤1000	≤1	≤32	>6	≤0.5	≤2	16	0.5	≤0.12	0.03	≤0.03	1
21	Lactococcus lactis	≤2	≤0.5	≤2	≤1	>4	≤0.5/9.5	1	≤2	≤0.25	0.5	≤0.12	0.25	≤0.25	0.5	0.25	≤0.25	≤0.5	≤1000	≤1	≤32	>6	≤0.5	≤2	8	0.5	0.25	≤0.015	≤0.03	1
22	Lactococcus garvieae	>16	≤0.5	4	2	>4	4/76	4	<2	>4	>4	1	1	0.5	1	0.25	≤0.25	>2	≤1000	≤1	≤32	>6	1	8	4	32	0.25	8	0.06	≤0.12
23	Lactococcus lactis	4	≤0.5	≤2	2	>4	≤0.5/9.5	2	<2	≤0.25	1	0.25	0.5	≤0.25	1	≤0.03	≤0.25	≤0.5	≤1000	>2	>64	>6	≤0.5	≤2	4	≤0.12	≤0.12	≤0.015	≤0.03	1
24	Lactococcus lactis	4	>4	≤2	2	>4	≤0.5/9.5	4	≤2	>4	2	0.25	0.5	≤0.25	1	0.06	≤0.25	>2	≤1000	>2	>64	>6	≤0.5	≤2	8	1	0.25	>8	>16	2
1	Streptococcus salivarius_ssp_thermophilus	≤2	≤0.5	≤2	≤1	≤0.5	≤0.5/9.5	≤0.5	≤2	≤0.25	≤0.25	≤0.12	≤0.06	≤0.25	1	≤0.03	≤0.25	≤0.5	≤1000	≤1	≤32	≤6	/	/	/	/	/	/	/	/
2	Streptococcus salivarius_ssp_thermophilus	≤2	≤0.5	≤2	≤1	≤0.5	≤0.5/9.5	≤0.5	≤2	≤0.25	≤0.25	≤0.12	≤0.06	≤0.25	0.5	≤0.03	≤0.25	≤0.5	≤1000	≤1	≤32	>6	/	/	/	/	/	/	/	/
3	Streptococcus salivarius_ssp_thermophilus	≤2	≤0.5	≤2	≤1	≤0.5	≤0.5/9.5	≤0.5	≤2	≤0.25	≤0.25	≤0.12	≤0.06	≤0.25	1	≤0.03	≤0.25	≤0.5	≤1000	≤1	≤32	≤6	/	/	/	/	/	/	/	/
4	Streptococcus equinus	≤2	≤0.5	8	≤1	≤0.5	≤0.5/9.5	≤0.5	≤2	≤0.25	≤0.25	≤0.12	0.12	≤0.25	1	0.06	≤0.25	≤0.5	≤1000	2	≤32	≤6	/	/	/	/	/	/	/	/
5	Streptococcus salivarius_ssp_thermophilus	≤2	≤0.5	≤2	≤1	≤0.5	≤0.5/9.5	≤0.5	≤2	≤0.25	≤0.25	≤0.12	≤0.06	≤0.25	1	≤0.03	≤0.25	≤0.5	≤1000	≤1	≤32	≤6	/	/	/	/	/	/	/	/
6	Streptococcus salivarius_ssp_thermophilus	≤2	≤0.5	≤2	≤1	≤0.5	≤0.5/9.5	≤0.5	≤2	≤0.25	≤0.25	≤0.12	≤0.06	0.5	1	≤0.03	≤0.25	≤0.5	≤1000	≤1	≤32	≤6	/	/	/	/	/	/	/	/
7	Streptococcus salivarius_ssp_thermophilus	≤2	≤0.5	≤2	≤1	≤0.5	≤0.5/9.5	≤0.5	≤2	≤0.25	≤0.25	≤0.12	≤0.06	≤0.25	1	0.12	≤0.25	≤0.5	≤1000	≤1	≤32	>6	/	/	/	/	/	/	/	/
1	Leuconostoc mesenteroides	≤2	≤0.5	4	2	1	1/19	≤0.5	≤2	≤0.25	≤0.25	1	0.25	>32	2	≤0.03	≤0.25	≤0.5	≤1000	≤1	64	>6	/	/	/	/	/	/	/	/
2	Leuconostoc mesenteroides	4	≤0.5	≤2	≤1	1	2/38	≤0.5	≤2	≤0.25	≤0.25	0.5	0.25	>32	2	≤0.03	≤0.25	≤0.5	≤1000	2	>64	>6	/	/	/	/	/	/	/	/
3	Leuconostoc pseudomesenteroides	8	≤0.5	≤2	≤1	≤0.5	≤0.5/9.5	≤0.5	≤2	≤0.25	1	0.5	0.12	>32	1	0.06	≤0.25	≤0.5	≤1000	≤1	>64	>6	/	/	/	/	/	/	/	/
4	Leuconostoc mesenteroides	≤2	≤0.5	≤2	≤1	≤0.5	2/38	≤0.5	≤2	≤0.25	≤0.25	0.5	0.25	>32	1	0.06	≤0.25	≤0.5	≤1000	2	64	>6	/	/	/	/	/	/	/	/
5	Leuconostoc mesenteroides	4	≤0.5	≤2	≤1	>4	1/19	2	≤2	≤0.25	≤0.25	0.5	0.25	>32	1	0.06	≤0.25	≤0.5	≤1000	2	>64	>6	/	/	/	/	/	/	/	/
6	Leuconostoc mesenteroides	4	≤0.5	≤2	≤1	1	2/38	≤0.5	≤2	≤0.25	≤0.25	0.5	0.25	>32	2	≤0.03	≤0.25	≤0.5	≤1000	2	>64	>6	/	/	/	/	/	/	/	/
7	Leuconostoc pseudomesenteroides	4	≤0.5	≤2	2	1	1/19	1	≤2	≤0.25	1	0.5	0.25	>32	2	≤0.03	≤0.25	≤0.5	≤1000	2	>64	>6	/	/	/	/	/	/	/	/
8	Leuconostoc mesenteroides	≤2	≤0.5	≤2	≤1	1	1/19	≤0.5	≤2	≤0.25	≤0.25	0.5	0.25	>32	1	≤0.03	≤0.25	≤0.5	≤1000	≤1	>64	≤6	/	/	/	/	/	/	/	/

CHL: Chloramphenicol; DAP: Daptomycin; GEN: Gentamycin; LZD: Linezolid; RIF: Rifampicin; SXT: Trimethoprim/Sulfamethoxazole; SYN; Quinupristin/Dalfopristin; TET: Tetracycline; ERY: Erythromycin; OXA: Oxacillin; AMP: Ampicillin; PEN: Penicillins; VAN: Vancomycin; LEVO: Levofloxacin; TGC: Tigecycline; MXF: Moxifloxacin; CLI: Clindamycin; STR: Streptomycin; CIP: Ciprofloxacin; NIT: Nitrofurantoin; FOXS: Cefoxitin; KAN: Kanamycin; NEO: Neomycin.
Not applicable (/).
